# Collectanea Medica: Consisting of Anecdotes, Facts, Extracts, Illustrations, &c.

**Published:** 1825-08

**Authors:** 


### COLLECTANEA MEDICA:

CONSISTING OF

### ANECDOTES, FACTS, EXTRACTS, ILLUSTRATIONS, &c.

***Relating to the History or the Art of Medicine, and the Collateral Sciences.***

Floriferus, ut apes, in saltibus omnia libant,

Omnia nos, itidem, depascimur aurea dicta.

We have much pleasure in giving insertion to this very interesting document of Sir Thomas Maitland; and we conceive that the circumstance of its having been written by a non-professional person materially enhances the value. We have lately given publicity to some well-written Essays on the other side of the question, and therefore seize, with the greater readiness, the present opportunity of illustrating the old proverb of "*audi alteram partem*."

Art. I.—*Copy of a Despatch from* Lieutenant-General the Right Honourable Sir Thomas Maitland, *to the* Right Honourable the Earl Bathurst, k.g., *dated Corfu,* 8*th of April,* 1819, *on the Subject of the Plague.*

My Lord,—As it appears that there is a question brought before Parliament relative to Quarantine Laws, I am led to apprehend that some doubts are entertained on the nature of the disease called the Plague, and on the means by which this fatal disorder is communicated.

As this is a subject to which I have had occasion to pay great attention, and one on which I certainly have had more practical experience than almost any other person, having arrived at Malta in the middle of the plague, and having since then witnessed the beginning and the end of three different attacks of this disorder,—one in the island of Gozo, one in the island of Corfu, and one in the island of Cephalonia, I feel it my duty to communicate to his Majesty's government the result of my observations on the nature and character of this malady.

The subject is in itself most important; for most certainly the treatment of plague, under the ancient system, is attended with a degree of unparalleled cruelty and tyranny, which can only be defended on the principle of positive and ascertained necessity.

This system cuts up by the root all those feelings of domestic life which are peculiarly endeared to the mind of man in a moment of sickness and distress,—rends asunder all the usual bonds of society,—and places the unfortunate patient in a situation of the most desolate isolation, at the moment when the only remaining comfort of life exists in the kindness of natural friends and connexions.

The quarantine-law, too, in the instance of plague actually existing, is not only most arbitrary in itself, but indefinite as it is arbitrary; and the whole circumstances attached to it are so revolting to the feelings, that I apprehend this is one of the causes why, in almost every instance, this fatal malady has been allowed to arrive at a great height before it is even declared to be plague. In the two great instances of the plague at Messina and at Marseilles, we accordingly find that no reliance was placed on its being the plague, till it had got to that dreadful head that occasioned those miserable scenes that ensued.

To a considerable extent, the same was the case at Malta, at the breaking out of the plague; and no man who has witnessed it can be surprised that the last rays of hope must expire before any society can submit patiently to a system of discipline, which may justly be regarded as an evil inferior only to the plague itself.

Under the same system, the quarantine-laws, the object of which is to prevent the introduction of plague, are attended in all instances with evils of great magnitude; and most of all, in the very serious effects they invariably have on the commercial relations of different countries.

It would be most fortunate indeed, then, if it could be made out that the world had hitherto been mistaken with regard to the causes and origin of the plague. At the same time, however, the consideration of this subject involves so deep a game, that I am convinced no step will be taken in regard to it without the most mature deliberation, and without the most positive proof. And I shall fairly own to your Lordship, that I am so thoroughly convinced, not only of the whole truth of the old system, but of the positive and absolute necessity of adhering to it, that it is with this view alone, and to impress it on your Lordship's mind, that I now venture to address you on the present occasion.

The whole subject resolves itself into this question, *whether the plague be acquired by contagion or infection?* But, before entering on it, it is absolutely necessary that we should come to a perfect and clear understanding of the meaning of these two words.

In many publications they are not defined at all; in some they are most strangely confounded; and in not a few they are altered exactly as suited the argument of the individual at the moment.

I beg it, however, to be completely understood, that, when I speak of infection, I mean that class of disease to which a particular temperature of the atmosphere, or some local cause, may give to the human frame a predisposing tendency.

When I speak of contagion, I mean distinctly to limit myself to that disease of which I am now treating; for, though there are others which may be brought under the same head, such as the small-pox, &c., still it does not appear to me, from the view I take of the case, that including them can answer any purpose whatever, as it is not my intention to enter on any theoretical or medical discussion on the character or nature of contagion or infection, but to limit myself entirely to facts. Those facts, which I am ready and able to prove, must lead every impartial person to concur with me in opinion that the plague is acquired only by contact; and that, therefore, the treatment which has hitherto been followed is the only mode of averting this dreadful calamity.

I carry my opinion on this head so far, that I have always held, and still hold, that medical advice respecting the preventive treatment of plague, so far from being useful, is almost invariably attended with evil consequences; for I have rarely met with any medical practitioner who had not some favourite theory to which he was attached, and by which he was so biassed, that he always endeavoured to bend the facts connected with the causes and appearances of the plague to this theory: hence he never contemplated it, in the only view in which I consider it, as the direct visitation of God, respecting the original cause of which we are hitherto ignorant, though we may arrest its progress by measures accompanied by experience, that have been followed for centuries, and of the beneficial effect of which I think I can bring forward the most incontrovertible proofs.

What new publications may have appeared on the subject, I know not: I have seen none but one by a Dr. Maclean, who was sent to Constantinople by the Levant Company; and, with regard to it, I can only say that there is not a single instance quoted in it, which came within my knowledge, that is not most strangely perverted, and most unfairly stated.

There is, indeed, on the whole of this subject, one great difficulty,—that is, the ascertaining of the fact. The country from which it generally proceeds—I mean Turkey—is under such rule, that we have none of those data to proceed on, which are so essentially necessary for the full and fair understanding of the question. Indeed, the information we can derive from that quarter is so loose, that every individual who writes upon the subject may find cases sufficient to support his peculiar view of the subject; and we cannot, from the returns of the government or police of that country, ascertain how far such cases are exceptions to a general rule, or fall within the general rule itself.

In the instances, however, which I am about to quote to your Lordship, we are relieved in a considerable degree from this difficulty; for, though I am far from saying that the returns I have had relative to the various plagues I have seen are accurate, or that such returns ever can be accurate, yet I think I am perfectly borne out, when I say they are more accurate than any others that ever have, or could have been exhibited.

I feel no inclination to enter into the causes which prevented the plague from being arrested at its commencement in Malta, or into its origin: it is unnecessary, and I was not there when it first appeared. As far, however, as I have been able to ascertain, I think I am warranted in stating that it was brought into Malta by a ship from Egypt, and conveyed out of that ship by a person smuggling some leather: this person and his family were the first sufferers from it. From some cause, it had proceeded to a great extent before any vigorous measures were adopted to arrest its progress: at length a strong executive police was formed by government, armed with the most ample powers to enforce, in the most peremptory and summary manner, those measures which, though for some time inculcated, had never been enforced.

The returns justify me in stating that, from the instant that this police was so established, the number of new cases gradually diminished, till the plague totally died away.

Whilst it was raging in Valetta, it made its appearance in several of the casals or villages in the country, where, from the pressure in Valetta till the police was established, no fixed measure was adopted for its suppression, and accordingly it raged in three of those with great violence, not more than four miles from Valetta.

As soon, however, as the good effects of the police establishment at Valetta were visible, it was wisely determined to carry the same measures into effect in each of these villages.

An officer was selected, who, with fifty men, was sent into the village of Zebug, where the disease had been particularly fatal, and in less than a month from their arrival the plague totally disappeared from that village. And it deserves to be specially remarked, that not one single soldier of the whole party was infected, though actually living in the village itself. About a fortnight after, a second trial was made: a similar detachment was sent into the village of Birchicara, when exactly the same result in every respect took place. Into the third village, viz. Curmi, troops were also sent in the same manner; but the situation of this village being in a very low swampy spot, remarkable for autumnal fever, the plague had proceeded to a very great length before the arrival of the troops, so that it required double the time for its extirpation here that was required in the other two villages; but here, too, none of the soldiers were attacked by it.

These are the principal facts relative to the plague at Malta, and they can all be proved by the most undeniable documents.

What, then, becomes of the doctrine of infection? The exclusive object of the troops was to prevent contact: this alone they were ordered to do, and this alone they accomplished. Every family was shut up in their own house, fed at their own doors, and sent into the lazaretto the moment the disease appeared. And, so far from the atmosphere being regarded as exerting any influence in spreading the plague, the inhabitants were forced not only to expose themselves, but every article of furniture, to the external air. The whole circumference within which the atmosphere might have had an effect did not comprehend above four miles: we have therefore this curious phenomena, so long as there was no police, the atmosphere being always of the same temperature, the disease uniformly increased; but I conclude that the advocates of non-contagion would contend, that the moment a regular police was established the temperature totally changed, and that the disease was shortly and finally eradicated.

The supporters, however, of the doctrine of infection maintain, that, with the decrease of the disease, its violence also subsides. To this alleged fact I paid the utmost attention; and I can prove, by authentic documents, that, so far from this being the case at Malta, the last cases were the most violent, and, in the instance of the last hundred, by far the most fatal.

I therefore, certainly, at that time was convinced that the only effectual mode of treating plague, was to act on the supposition of its being contagious. Of this I think I can give your Lordship a pretty convincing proof, —and a proof, too, that must convince any man whose mind is not biassed on this subject. I proclaimed that the plague was completely at an end, and allowed the whole population at Valetta to mix together, the very day that a strong case of plague had occurred within a mile of that city. Now I think you will agree with me, that, if it had been an infectious disease (in my understanding of the word), nothing short of perfect insanity could plead an apology for any man to declare that no new case of plague would occur. So convinced was I, however, of the firmness of the ground on which I was proceeding, that I issued this proclamation at the very moment I was driving into the lazaretto infected people from its very neighbourhood; and it is undoubtedly true that, as I proclaimed it, so it happened; for no new case of plague occurred after I had granted free pratique.

I think I should be now authorised to draw the natural inferences from these facts; but I shall defer doing so till I have apprised your Lordship of the circumstances attending the other three cases of plague which have fallen under my particular observation.

I shall pursue this course; for, though I think I have incontrovertible grounds to go upon, even limiting myself to what I have already stated, still your Lordship will find that I am fortified in all my opinions by what I have yet to advance, to such a degree, that my opinions, I conceive, cannot be successfully attacked.

At Gozo, the circumstances attending this disease were as follow; and I think your Lordship will agree with me, that we are much more likely to get at the real character of the disease when it is confined within a small circumference, and when we have time and means to attend to its commencement, its progress, and its end, and to follow all its ramifications in the minutest manner, than when it has taken deep root, and we are so overpowered with the extent of the calamity, that our observations must be more vague and general.

Either on the 2d or 3d of March, 1814, a report was made to me that a case of fever had happened at Gozo, which was of a suspicious nature. I immediately ordered it to be treated as plague, till it was ascertained satisfactorily whether it was this disease or not.

This order was, of course, immediately carried into effect: unfortunately, however, the weather was so violent, that, for the subsequent four or five days, we could have no communication with Gozo. On the 7th of the same month, however, I received information that there was no doubt but it was the plague; and, though I should not be able to prove it in a court of law, yet I am perfectly convinced that it was introduced into Gozo in the following manner:—

When the cordon of troops was first drawn round Curmi, (the third village I mentioned when treating of Malta,) it embraced a space of fully a quarter of a mile on all sides beyond the village itself, and this space was nearly covered with small houses and gardens. In these the plague had made its appearance, and, of course, the first operation was to expurgate them, and send the suspected people to the lazaretto, that we might gradually be enabled to diminish the extent of the cordon, and draw it close round the village itself. This operation was accordingly carried into immediate effect.

It appears, however, that one of the persons who had been sent into the lazaretto for forty days, on his being liberated, directly proceeded to his house, which had been originally within the cordon, but which was now without it, in consequence of its having been straitened, as above mentioned.

This person, before he went into the lazaretto, had concealed in his garden a small box: on quitting the lazaretto, he dug it up, carried it to town, and then immediately proceeded with it to the island of Gozo, where he had some relations in the village in which the plague afterwards appeared. He then opened it, and gave what they term a faldetto (a black silk cloak, universally worn by the women of Malta,) to his relation. I have not the smallest doubt that the plague was by this means generated in Gozo.

As soon as I received the report of the 7th of March, already mentioned, I lost not a moment in sending a detachment of soldiers, (who, from their experience at Malta, knew in what manner to treat the plague,) with the strictest orders to enforce the system I had there pursued. By this time the plague had appeared in five or six houses in the neighbourhood of that in which it originally broke out, and within this circumference it was immediately confined. Unluckily, however, the commanding officer, contrary to his orders, employed (I believe from necessity,) a few days afterwards, a guard of the island militia, instead of regular troops, consisting of seven men. One of these, being a relation, had communication with the infected houses, caught the plague himself, and communicated it to the rest of the party; and these communicated it to their families.

We were, therefore, obliged to begin anew: these families were immediately seized and confined in the lazaretto. Thus its further progress was effectually stopped, and the plague at Gozo ended.

Now, how this can have any thing to do with infection, I am totally at a loss to discover. The plague appeared at the very best season of the year, and the village itself lies very high; we traced every one who had the plague from beginning to end; there was no difficulty in ascertaining how they got it, and as little difficulty in stopping it when it was traced.

We held, from first to last, that it was propagated by contact; that it was stopped by preventing that contact; and I here again proclaimed that the plague was at an end, and so it turned it.

At Corfu and Cephalonia, the circumstances relative to the plague were as follow:—

And first, with respect to Corfu, I feel it my duty to state to your Lordship the whole of my conduct on the occasion, without reference to any man, or consideration of any kind.

The plague broke out in this island before I arrived in it, at the beginning of 1816; but the circumstances connected with its origin were exactly these:—

A vessel came into Malta from Corfu, which brought me letters from Lieut.-General Campbell, and I think to the admiral also: they stated that nothing new had happened. But, about an hour afterwards, Dr. Grieves, sub-intendant of quarantine at Malta, called upon me, and asked me if I had heard from Corfu? to this I answered, I undoubtedly had, but that I had no new information of any kind. Dr. Grieves then pulled out of his pocket a letter to him from a physician at Corfu, which letter I suppose can be now produced; but the substance of it was so extraordinary, that I immediately called a meeting of all those persons who I thought had most experience in the plague, and read to them this letter from the physician; putting it to them whether, from their experience, what was therein stated did, or did not, prove that the plague actually existed in Corfu.

They unanimously agreed that it was the plague; and, if I recollect right, the terms in which I wrote to General Campbell (though he was not then under my orders, and I had no authority to give him more than my opinion on the subject,) were, that he must consider it as the plague, and nothing else but the plague.

It happened to me, for reasons of which your Lordship must be well aware, to proceed immediately after I received the above-mentioned letter, —viz. in February 1816, to Corfu; and I then found, to my great surprise, that, instead of the plague having been declared in Corfu, it was almost a proscribed idea that the plague existed there at all.

I was, of course, obliged to take my own measures, and, having carefully examined every thing, and inquired into every preceding circumstance, I had no difficulty in declaring at once my opinion that it was the plague, and nothing but the plague; and I gave orders here, as I had done elsewhere, that not a nominal, but a real and most strict perseverance, should be maintained in those rules which I had hitherto seen effectual in arresting the disease.

Your Lordship will, however, perceive that I was here placed in a very different predicament from that in which I had stood in regard to Gozo or Malta; for there I could select my men and my officers for this service.

In the lower district of Corfu, viz. Leftimo, the troops had already been all sent in: none of them in the way I should have employed them; and taken generally from a very irregular return and description of troops, no attention having been paid to the character of the officers or men; and, of course, therefore exposed to communication with the persons in the infected districts.

Upon this part of the subject I feel disinclined to press what passed upon your Lordship; but, if there be a question upon it, I call upon Lieutenant General Campbell to produce to me the last letter he received from the physician, then acting at the head of the medical department in the infected district: stating how sorry he was to see the frigate, which was conveying me to Corfu, arrive that night, because he thought that, if she had not appeared for two days, the disease would have been completely eradicated.

Having, however, sent for and questioned the physician, and examined the thing myself, I ascertained that no man was permitted to say any thing of the plague, because no such thing existed. I declared immediately by proclamation that the plague was in the island,—that no person could doubt it,—and that no human being could avert the dreadful consequences, unless the people themselves lent their aid.

I then put in practice my own system; but I am disinclined to use the word system, lest it might imply that I have some theory in my head, like the doctors. The result of my plan or system was, that, instead of the disease disappearing after I arrived, as the physician expected, with the assistance afterwards afforded me by Major-General Philips, who had seen the whole of the business at Malta, and displayed throughout a great degree of ingenuity and talent in supporting my measures, it took me four months to get rid of it, under circumstances of a most advantageous nature. All the documents relating to this part of the subject are in existence.

When the plague was fairly exterminated, I issued a proclamation to that effect, and nothing from that moment like the plague has ever shown itself in that island.

It now becomes necessary for me to explain to your Lordship how the plague was introduced into Corfu; and I have no difficulty in stating that I have legal proof, such as would convince any jury or judge in any court of justice.

For many months, great doubts were entertained in what manner the plague was introduced. There are reports of such an atrocious nature, that I shall not name them to your Lordship: they attacked at once the character of the British government and the reputation of my predecessor. Such reports could only have proceeded from disappointed political views.

The case, however, was strictly this:—A boat had sailed from the upper part of the Adriatic, called at Parga, and reached the district of Leftimo, in Corfu, on a smuggling concern. This concern consisted of two boxes of goods; one of which contained a parcel of little round red caps, which the Greeks are obliged to wear as an emblem of Ottoman supremacy: this was not opened.

The person who had the property in this boat, resided a few days in a village of this district, when his wife, or the woman with whom he cohabited, died.

It is quite clear, whatever was the nature of the disease of which she died, it had no effect whatever with regard to the country; but the man immediately went to a house called the Casa Polita, where he had lodged his box, and he then pledged it for a certain consideration, to be repaid in six months; and, if he did not redeem it, the box was to become the property of the person who advanced the money.

At the expiration of this time, the man who had purchased this box opened it in the presence of a person who lived in a neighbouring village; five or six other persons were also present.

An immediate alarm took place; for seven, eight, or nine persons, who had been engaged in smuggling, were all taken ill in the Casa Polita. The whole of the family where the person just alluded to lived, together with himself, died. The belief was, that sorcery existed, and that the house must be exorcised.

Accordingly the papas of the different villages were all called in, and having exorcised the house they went away, and each and all of them carried into their several villages the plague.

In consequence of this, it spread, after my arrival, to twenty-six or twenty-seven different villages; but the measures I adopted, seconded as they were by the assiduity of Major-General Philips, enabled me to declare, under circumstances particularly unpropitious, that the plague at Corfu had ceased; and from that hour to this we have (thank God!) been perfectly clear of it.

I have now to state to your Lordship how we learnt the circumstances attending the plague at Corfu.

Our information came from the sole survivor of the Casa Polita, a young girl about fifteen years of age, who had had the plague, but recovered: after having gone through various quarantines, she came forward, and told what I have just stated to your Lordship. She was examined and cross-examined; every evidence relating to collateral facts was produced: the conviction on my mind, and I believe on that of every person who examined the case, was and is, that her story was true; nor, indeed, is it now attempted to be denied.

The day after I had proclaimed pratique, I sailed, under your Lordship's directions, for England; but, as I wished to call at Cephalonia, the man-of-war, on board of which I was, anchored at Argostoli. I there learned that the plague was in Cephalonia: on this I sailed for Zante, and directed that information should be sent to me there, and that measures should be immediately adopted for arresting the progress of the disease at Cephalonia.

I arrived at Zante the same day, and, receiving the information, I immediately ordered, as far as I can now recollect, about fifteen persons, (some of whom I named, but all of whom I knew were acquainted with the business,) to be instantly sent to Cephalonia, and I put the whole under Colonel Rivarola, who had been employed, during the whole business at Malta, as head of the police there, and, at the time I am speaking of, was temporary commandant at Zante.

Though I am not informed of the exact detail, I can pledge myself that the same measures were adopted, and that the same result took place at Cephalonia as at Gozo; and that in a very short space of time the plague ceased there, and its termination was proclaimed accordingly.

This is the short but true history of the four plagues, of which I have been witness; the whole of which have passed under my observation and superintendance, been treated according to my own opinions and judgment, and declared to be extinguished, according to my own belief.

It is proper, however, to state that I did not arrive at Malta till after the police had been established in Valetta, and the number of new cases had daily very considerably diminished.

From first to last I held then, as I hold now, that the disease could be acquired only by contact; and all the documents, proceedings, precautions, and proclamations, which are voluminous, tending to one single point,—viz. to prevent contact and the effect of contagion, can be transmitted, if necessary, to your Lordship.

These documents go infinitely deeper into the history of plague than any other documents on the subject I have ever read; and I must say that, in my opinion, the single fact of having, in four different plagues, proclaimed that each was at an end, and after such proclamation not one case, up to the present moment, has occurred,—not even a case in which plague could be suspected,—in any of the four instances, says more in favour of the doctrine I maintain, than all the arguments and reasonings that can be adduced on the subject.

I proclaimed its extinction at Malta upwards of five years ago,—at Gozo, upwards of four years ago,—at Corfu, two years and ten months ago,—and at Cephalonia, about two years and a half ago.

But I have gone further; for I have stated to your Lordship the causes of the introduction of the plague in three of these instances; and though, undoubtedly, it may be alleged that the proof upon this is not quite certain, still I hold it to be as much so as any evidence you can ever get upon any such subject.

I have invariably found that preventing contact stops the disease, and, that, so long as contact is permitted, it uniformly increases. Against these facts (and I defy any man to controvert them, supported as they are by documents written and published at the moment,) it has been stated that this is a disease acquired by infection, and not by contact. I beg leave, however, for one, to say, that I have never seen any thing, or heard any thing, that carried with it to me any conviction upon this head.

There is usually an attempt made,—but I presume such attempt must now be given up,—to prove that which is called the yellow fever in the West Indies and in America, and that the fever which prevailed at Gibraltar, were all subject to the same laws as the plague; that is to say, that they originated in contagion or infection, as suited the system of the individual who was giving his opinion.

It is, I believe, now generally admitted that these three classes of fever were not subject to the law of contagion, as I have stated it; but that each of them is subject to the laws of infection, and that they are liable to recur at each of the three places above mentioned, at particular seasons of the year, notoriously known to be the most unhealthy seasons in the West Indies, America, and Gibraltar.

It has been frequently attempted to prove the same with respect to the plague, and it is certainly necessary that it should be made out, if the doctrine of infection is supposed to apply to it; and we accordingly find that, whether it be in Egypt, at Smyrna, or Constantinople, an effort has been made to convince the world that it stops in these places at certain seasons, and commences at others. For my own part, I can only say that he would be a bold man who would venture to assert that the plague ever was eradicated at any season of the year, in any one of the three places just mentioned: on the contrary, I hold nothing more certain than that it constantly exists in them all, showing itself with greater violence at one time than another, and hardly taken notice of till it arrives at such a height as to be seriously alarming to the whole community.

Let us see, however, how this doctrine applies to the four plagues, of which we have much better proof of all the facts attending them, than of any other that ever had existence.

It broke out at Malta in May, the very healthiest season of the year; it began to decline in August and September, after the police was formed, decidedly the most unhealthy months of the year, and was not totally extirpated till the month of February following.

At Gozo, it began in March, the very healthiest season of the year, and was extirpated precisely before commencement of the violent heats, which is the unhealthy season.

But the case of Corfu, in this respect, is still stronger: the plague began here in November, with the healthy season; it was declared at an end exactly as the unhealthy season began. And what was the case with regard to this? The case was, that the troops employed in stopping the plague, and who, generally speaking, never caught it, did suffer heavily after the plague was over, from the common fever of the country, in consequence of our being forced to keep them in unhealthy stations after the most unhealthy season had commenced.

At Cephalonia, the plague began and ended during the hot months; and (which I forgot to mention before) was notoriously brought into that island by persons who had been reaping the harvest on the continent, in Epirus, or in the Morea.

This statement cannot be contradicted; as we have the plague in these four instances evidently showing itself, and proceeding in its career, without reference to its ceasing with the healthiness or the unhealthiness of the climate. It was stopped in each of these four cases by means totally foreign to the treatment of infectious diseases,—means quite peculiar to itself; and this treatment did effectually stop it.

The advocates of the doctrine of infection, however, are disposed to place this point on a footing which can never be completely proved to be unfounded, by holding that all this statement may be true, but that still the plague may be subject to the law of infection; because no man can say precisely where there may be a predisposition in the atmosphere to give a disease, or when such predisposition may cease: this is undoubtedly true.

It is impossible, then, to give any direct proof that it is got exclusively by contact, because no one can prove the exact tendency of the atmospheric air at the moment; but we have this complete and triumphant answer to make to all such doctrines, that, though I cannot try to prove what, in the nature of things, it is impossible to establish by evidence, I will show your Lordship that, on the supposition that contact alone can give it, it has uniformly stopped under every circumstance of atmosphere. I must, therefore, think that I do not assume too much, when I state that the sound doctrine on the subject is this,—that, if the absence of contact stops the plague, the allowing of contact must be the real means of producing it.

But upon this point also a modification is always introduced,—viz. that, allowing contact to give it, is it, or is it not, possible that the state of the atmosphere may render it of a very different character in some seasons than in others. Upon this head I do not feel disposed to enter into any discussion. I am willing to concede the possibility of this, though the fact is otherwise, as far as my experience goes; but all that I am now contending for is with regard to the treatment which does stop the plague, and not with regard to any local or particular circumstances that may increase or diminish the violence of it; and the whole I contend for is this:—that I can bring the most positive proofs that want of contact stops it; and I defy any man, or set of men, to bring equally convincing proofs that, treating it as an infectious disease, it will ever stop, till it exhausts itself by the death, dispersion, or destruction, of the community where it exists.

As a further proof of this, I need only bring forward the case of the troops which I employed.

In the few instances that occurred, and they were extremely few, it was uniformly observed of each soldier that took the plague, that he was loose in his conduct, and neglectful of the necessary precautions. Those, on the contrary, who attended to these precautions, never took it. They were sent into several villages, many of them with streets but a few feet wide; they did the severest night-duties of all kinds in these villages; they lived in exactly the same atmosphere as the inhabitants; yet they never caught the disease, though it was raging in the villages. They were stationed within a yard or two of camps and hospitals, in which the plague was raging with great violence, and they never caught it. And, lastly, they were exposed to all those hard duties, which, in all infectious diseases, are known to give a predisposition to the most violent and fatal type of the prevailing disease, and yet they never caught the plague.

I know not what strong and direct proof is, if this be not such, that the plague is got by contact, and not by infection; and of each and all these facts I am ready to send your Lordship the most complete and perfect evidence.

I do not, however, mean to assert, that in all cases, and under all circumstances, contact will infallibly produce the plague, for there are certain strong instances to the contrary. Neither do I hold that there are not instances of infectious diseases that may be got by contact. But the general position I maintain is this,—that plague is got by contact, and infectious diseases by the state of the atmosphere, or some other local cause; and that whatever instance may be quoted to the contrary, is an exception to the general rule.

As I have expressly stated that, in my opinion, the fever at Gibraltar was infectious, it may be alleged that I have given an opinion upon those medical disputes which have recently occurred, how far that fever was contagious or infectious. This, however, is foreign to my intention, as well as my situation. My observations apply to infection of all kinds, whether generated by the state of the atmosphere, or by any other local cause,—including in this last term many species of what may be called by some contagion, arising from any communication but that of direct contact, or, in other words, actually touching the body of an individual, or any substance which is impregnated with the cause that produces plague. Neither shall I say one word to your Lordship on the medical treatment of the plague, further than to express my regret that, as far as I am aware, (and I have had a great deal of conversation on the subject,) we are just as much in the dark, in respect to any cure for this terrible disease, as we were at the moment it broke out at Malta.

I shall do myself the honour, in a future letter, to state to your Lordship my further sentiments on this interesting subject, and particularly what I think may be done with safety in altering the old system; and I shall then submit to your Lordship a few observations on that system, the inconvenience which attends it, and the possibility of remedying that inconvenience, as far as may be in any way consistent with prudence.

I have the honour to be, &c. &c. &c.

(Signed) T. MAITLAND.

